# Imaging Mass Spectrometry-Based Proteomic Analysis to Differentiate Melanocytic Nevi and Malignant Melanoma

**DOI:** 10.3390/cancers13133197

**Published:** 2021-06-26

**Authors:** Rita Casadonte, Mark Kriegsmann, Katharina Kriegsmann, Isabella Hauk, Rolf R. Meliß, Cornelia S. L. Müller, Jörg Kriegsmann

**Affiliations:** 1 Proteopath GmbH, 54926 Trier, Germany; joerg.kriegsmann@dp-uni.ac.at or; 2 Institute of Pathology, University Hospital Heidelberg, 69120 Heidelberg, Germany; Mark.Kriegsmann@med.uni-heidelberg.de; 3 Department of Hematology Oncology and Rheumatology, University Hospital Heidelberg, 69120 Heidelberg, Germany; Katharina.Kriegsmann@med.uni-heidelberg.de; 4 Faculty of Medicine/Dentistry, Danube Private University, 3500 Krems-Stein, Austria; isabella.hauk@hotmail.de; 5 Institute für Dermatopathologie, 30519 Hannover, Germany; institut@dermpath-hannover.de; 6 MVZ für Histologie, Zytologie und Molekulare Diagnostik Trier, 54296 Trier, Germany; prof.mueller@patho-trier.de

**Keywords:** classification, imaging mass spectrometry, MALDI, melanoma, nevi, proteomics

## Abstract

**Simple Summary:**

Histopathological, immunohistochemical and molecular investigation of melanocytic lesion is the standard for the diagnosis of melanocytic lesions that are difficult to classify. To ensure correct diagnosis, mass spectrometry has been proposed, specifically in the differential diagnosis of spitz nevi and melanoma. Imaging mass spectrometry is an evolving technology, able to discriminate various tumor entities, which combines morphological features and mass spectrometry. The aim of this study is to apply imaging mass spectrometry to melanocytic lesion to discriminate melanoma from nevi.

**Abstract:**

The discrimination of malignant melanoma from benign nevi may be difficult in some cases. For this reason, immunohistological and molecular techniques are included in the differential diagnostic toolbox for these lesions. These methods are time consuming when applied subsequently and, in some cases, no definitive diagnosis can be made. We studied both lesions by imaging mass spectrometry (IMS) in a large cohort (*n* = 203) to determine a different proteomic profile between cutaneous melanomas and melanocytic nevi. Sample preparation and instrument setting were tested to obtain optimal results in term of data quality and reproducibility. A proteomic signature was found by linear discriminant analysis to discern malignant melanoma from benign nevus (*n* = 113) with an overall accuracy of >98%. The prediction model was tested in an independent set (*n* = 90) reaching an overall accuracy of 93% in classifying melanoma from nevi. Statistical analysis of the IMS data revealed mass-to-charge ratio (*m/z)* peaks which varied significantly (Area under the receiver operating characteristic curve > 0.7) between the two tissue types. To our knowledge, this is the largest IMS study of cutaneous melanoma and nevi performed up to now. Our findings clearly show that discrimination of melanocytic nevi from melanoma is possible by IMS.

## 1. Introduction

Melanoma is the fifth most common cancer among men and women according to statistics from the American Cancer Society’s (ACS) publication, Cancer Facts & Figures 2021 [1]. The number of people diagnosed with melanoma has been rising rapidly over the past few decades.

Approximately 25–33% of cutaneous melanomas arise from nevi [2,3], this percentage can increase up to 50% for high-risk patients presenting numerous nevi. Benign as well as atypical nevi have been shown to exist in histologic continuity with melanoma, suggesting that these melanocytic proliferations are also susceptible to malignant transformation [4]. However, there are melanocytic lesions with an ambiguous histopathologic appearance demonstrating some but not all of the features associated with common melanomas, making their classification difficult, with a certain interobserver variability among pathologists [5]. The exact diagnosis of melanocytic lesions is required for adequate therapy and prognosis. Besides histological evaluation by conventional histology and immunohistology [6,7,8], molecular pathological methods have been developed to aid the diagnosis [9,10,11]. Despite the application of next-generation sequencing, definite discrimination of melanomas from nevi may be challenging.

In addition to molecular pathological methods (DNA- or RNA-based methodology), proteomics has been considered a promising technology for discovering biomarkers. In recent years, several proteomic methodologies have been developed that now make it possible to identify, characterize, and comparatively quantify the relative level of expression of hundreds of proteins that are co-expressed in specific cell types or tissues [12,13,14]. The introduction of the imaging mass spectrometry (IMS) technique allows the investigation of peptides in a three-dimensional manner in histological specimens. IMS is a label-free analytical technique for the direct analysis of biological samples that revolutionized biological mass spectrometry, and especially proteomics, because it ionizes biomolecules, maintaining their spatial integrity. Recent advances in instrumentation, experimental procedures, and bioinformatics approaches have greatly improved IMS technology. For example, IMS analysis of large tissue sections (~4 cm^2^) at high spatial resolution was previously impaired by the prohibitively slow acquisition speed of existing platforms. Commercial instruments now incorporate lasers with the ability to operate with a speed of >50 pixel/second, allowing a measurement time of less than 1 hour even at small pixel size (≤20 µm). Fast data acquisition is now comparable to routine clinical analysis, such as immunohistochemistry, and will greatly help to increase sample throughput. New software is now available that allow statistical analysis of spectral data from several hundred thousand pixels; however, the need of a data reduction tools will become important. In addition, increased molecular accuracy for class prediction model has been reported in several proteomics studies using IMS; thus, molecular characterization of tumors for each patient individually can be achieved [15,16,17,18,19,20]. Other advances, such as combination of IMS data with other imaging modalities by using machine learning provide an opportunity to develop next-generation diagnostic tools [21]. The development of IMS technology in conjunction with conventional methods holds great promise for cancer protein marker discovery and clinical translation. Moreover, proteomic approaches have been used previously on melanoma samples to identify diagnostic and prognostic signatures [22,23,24,25,26].

In this study, we applied matrix-assisted laser desorption/ionization (MALDI) imaging mass spectrometry (IMS) to formalin-fixed paraffin-embedded (FFPE) tissue specimens of malignant melanoma and melanocytic nevi to generate a proteomic classification method capable to discern between both entities. One of the key aspects of MALDI IMS for routine clinical application is to develop sample preparation protocols that ensure consistent and accurate data acquisition. Sample preparation may vary between different tissues or types of experiment, and this may produce variations in the data and data analysis outcome. Thus, it is important to maintain controlled and standardized sample preparation procedures. We have recently assessed the quality and reproducibility of MALDI IMS on FFPE tissues in a multi-center study, which are prerequisites for clinical application. We could demonstrate that measurements are reproducible when using a standard protocol, including standard instrumentation and sample preparation conditions [27]. Here, we conducted preliminary experiments to optimize our routine sample preparation protocol on skin tissues in which experimental and data quality as well as reproducibility are assessed. We finally analyzed and classified all melanocytic tissues using the proposed protocol. To our knowledge, this is the largest proteomics-based MALDI IMS study conducted on benign and malignant melanocytic tissues.

## 2. Results

### 2.1. Sample Preparation Optimization

In a prior investigation, we explored two different antigen retrieval temperature and trypsin deposition conditions (wet, dry) for their effect on the peptide signal. These procedures are performed on serial sections from the same melanoma tissue in duplicate. We assessed the quality of data and the reproducibility of such methods by comparing peptide profiles obtained from 100 random spectra from each tissue and each protocol using R statistical software [28]. Finally, we performed a qualitative evaluation of the peptide peaks with regard to intensity and tissue distribution. The number of detected peaks between each protocol was similar for each replicate (Figure S1). However, the overall peak intensity increased considerably using the protocol with the antigen retrieval temperature = 110 °C (Figure S2). With this temperature condition, wet and dry trypsin deposition methods showed similar spectra profiles, although the wet approach was faster (~10 min) compared to the dry condition (~30 min). In addition, signal delocalization behavior across the measured areas did not appear to be a problem in any of the experiments (Figure S2). As a result, the antigen retrieval temperature condition at 110 °C with the wet trypsin deposition method was chosen for further analyses.

### 2.2. Statistical Analysis and Classification Study

**Figure 1:**
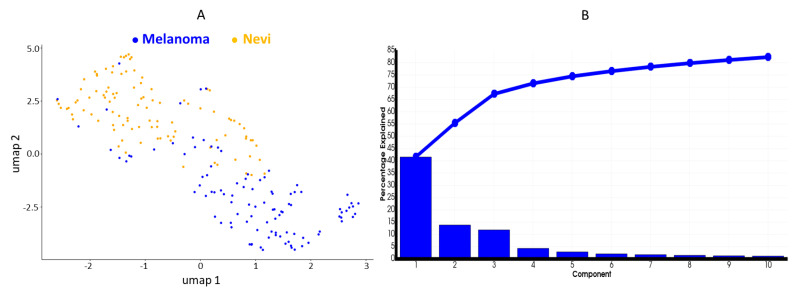
Dimensionality reduction of the imaging mass spectrometry (IMS) spectral data analyzed by SCiLS Lab and R statistical software. (**A**) Uniform Manifold Approximation and Projection (UMAP) two-dimensional (2D) plot of melanoma (blue spots) and melanocytic nevi (yellow spots). The results show a differential distribution of the two spot regions in the dataset. (**B**) Variance plot of the principal component analysis (PCA) showing the first three components containing approximately 70% of the variance percentage of the data.

Mass spectra from areas of interest were obtained for each sample including more than 300 ion peptide peaks. Uniform Manifold Approximation and Projection (UMAP) and principal component analysis (PCA) were initially used to simplify multidimensional data by combining similar or redundant information into fewer variables. UMAP visually revealed two distinct clusters of data corresponding to melanoma and melanocytic nevi. Following PCA the top 3 principal components accounted for nearly 70% of the overall variability (Figure 1).

**Figure 2:**
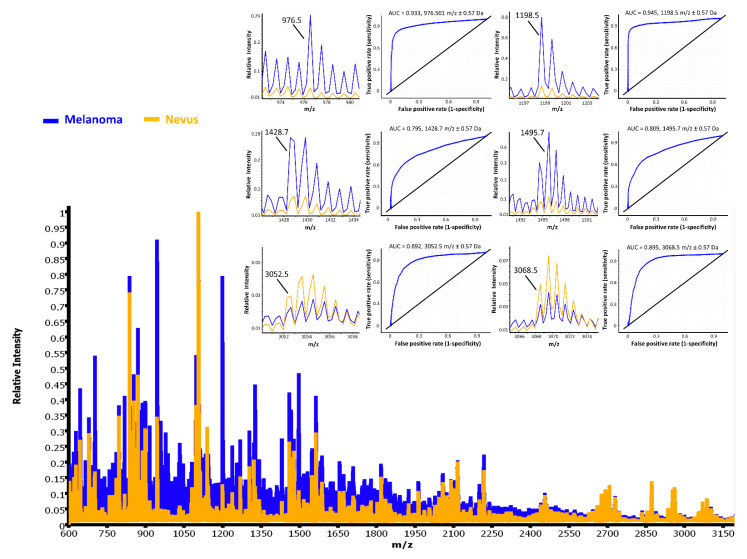
Overlay of the average spectra of melanoma (blue) and melanocytic nevi (yellow) in the training set. Zoomed spectra of some of the discriminating peaks are shown. Specifically, the *m*/*z* ratio, equal to 976.5, 1198.5, 1428.7, and 1495.7, were highly expressed in the melanoma, while signals at 3052.5 and 3068.5 *m*/*z* were overexpressed in the melanocytic nevi. Receiver operating characteristic curve (ROC) analysis is shown for each peak and was >0.76.

The diagnosis of the tissues was established by a combination of clinical information, histology and molecular pathology. Hematoxylin and eosin (HE) images of the same sections used for MALDI IMS showed very similar histological features to the HE serial section annotated by the pathologist. This allowed to correlate HE-stained areas of interest with the corresponding IMS data collected from the same section. Comparison of the mass spectra average profiles between benign and malignant tissues showed specific peptide expression patterns between the two tissue types. A significant change in expression of the *m*/*z* peaks was evaluated based on receiver operator characteristic curve (ROC) analysis, which revealed 187 ion peptides discriminating the two phenotypes (*p* ≤ 0.001, area under the ROC curve (AUC) > 0.76). A spectral view comparison of some significant peptides is shown in Figure 2.

From the proteomic signatures obtained, including 187 peptide peaks, a linear discriminant analysis (LDA) classification model of malignant and benign melanocytic tissues was generated and validated. Leave-one-out cross-validation was applied, which indicated a good performance of the prediction model (accuracy > 97%). The LDA model classified the training set with a sensitivity of 96% (54/56 melanoma correctly classified) and a specificity of 100% (57/57 nevus correctly classified). The algorithm was then run in the testing set, where 91% (41/45) of melanoma and 95% (43/45) of nevi patients were correctly classified (Table 1). The model achieved an overall accuracy of 98% in the training and 93% in the testing set.

**Figure 3:**
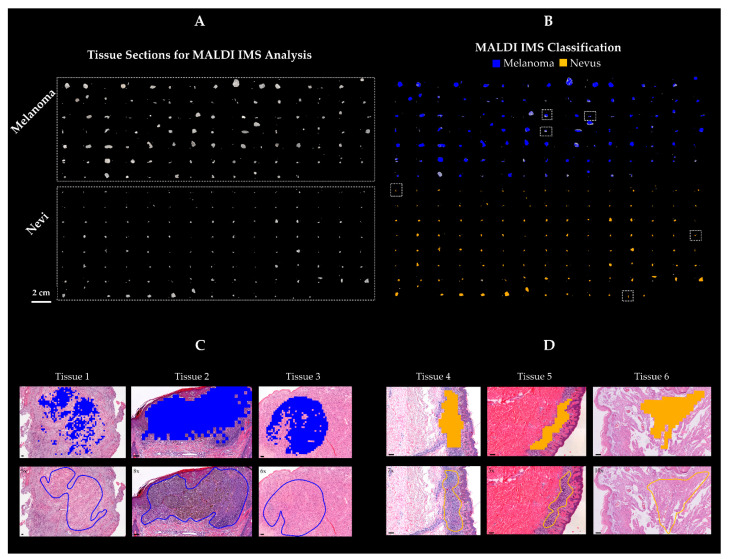
Imaging mass spectrometry classification results. (**A**) Cohort of melanoma (*n* samples = 101) and nevi (*n* samples = 102) tissues. (**B**) Statistical classification model (LDA) was applied to all tissues and visualized in a color-encoded representation. Specifically, blue was used for melanoma and yellow was used for melanocytic nevi classification. Class images resembled the histopathological diagnosis made by pathologists. Enlargement of the MALDI IMS classification result of some representative tissues (**B**, dashed square) is shown for three melanoma (**C**, Tissue 1, Tissue 2, Tissue 3) and three nevi (**D**, Tissue 4, Tissue 5, Tissue 6). MALDI IMS classification of melanoma (**C**, pixels in blue) and melanocytic nevi (**D**, pixels in yellow) is in agreement with the histopathological diagnosis (**C**, annotated regions in blue line = melanoma; **D**, annotated region in yellow line = nevi). Scale bar = 100 µm.

From the 187 *m*/*z* considered and based on the t-test statistical comparison (*p* ≤ 0.001), a set of 18 peaks was selected which showed a minimum two-fold intensity difference average. Specifically, 15 peptides peaks (*m*/*z* 631.4, 816.4, 872.4, 901.3, 914.4, 957.6, 958.5, 976.4, 1032.7, 1198.7, 1325.7, 1428.7, 1489.7, 1490.8, and 1495.7) were overexpressed in melanoma, while three peptides (*m*/*z* 1138.6, 3052.6, and 3068.4) were overexpressed in nevi. When we used this short peptide signature (18 peptide peaks) to classify the same cohort of samples, the overall classification accuracy in the training and testing set was 93% and 92%, respectively. Specifically, 49/56 melanoma and 56/57 nevi in the training set, and 39/45 melanoma and 44/45 nevi in the testing set were correctly classified (Table 1). Thus, each of the classification models showed a high recognition capability. The outcome of the classification can be easily made visible using the class imaging function in SCiLS Lab software, where class images were visualized based on a color-encoded representation (Figure 3). As a result, LDA classification was congruent with the histomorphological assessment.

**Figure 4:**
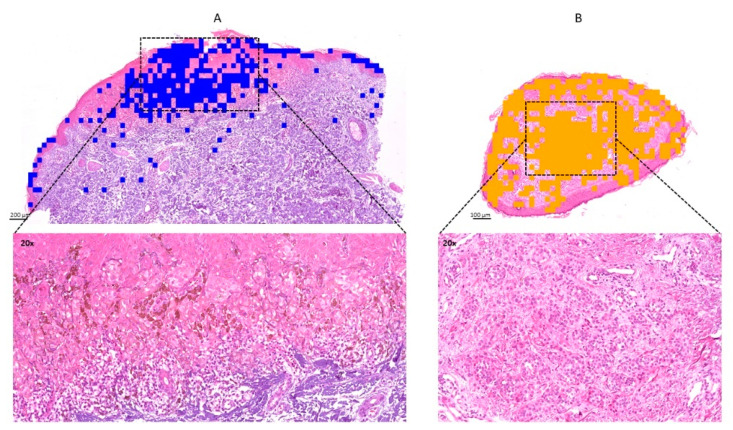
Correlation of imaging mass spectrometry (IMS) classification and histology. (**A**) Hematoxylin and eosin staining overlay with IMS classification (pixel spots in blue) of a melanoma sample. (**B**) Hematoxylin and eosin staining overlay with IMS classification (pixel spots in yellow) of a nevus case. Square dashed lines represent the pathological annotated areas that correlate with the IMS classification. Images at 20× magnification view are shown for malignant melanoma (**A**, down) and melanocytic nevi (**B**, down).

A representative correlation of IMS classification with the histology of a melanoma and a melanocytic nevus tissue is shown in Figure 4, depicting a strong association between the diagnosis and IMS classification. These tissues were independent and not included in the sample cohort for training and testing the classification model. In this example, a total of 34 spectra were included in the histological annotated area of melanoma, and 30/34 were classified correctly by IMS. Likewise, for the nevus sample, 63/66 spectra were classified as nevus in agreement with the pathological diagnosis.

### 2.3. Protein Identification

Eighteen *m*/*z* peaks were the most prominent discriminatory peptides in melanoma compared with benign nevi. In total, 11 out of 18 peptides were sequenced and identified directly from tissue using an on-tissue MS/MS strategy. Table S1 shows a list of the tryptic peptide masses with their corresponding identifications. A MASCOT search identified the peptide sequences with a lower mascot score of 51 as DNA-3-methyladenine glycosylase (*m*/*z* 1138.6), and the highest score of 168 as collagen alpha-1(I) protein (*m*/*z* 3068.4), which were both highly intense in nevi. Four *m*/*z* ions were identified as vimentin peptide ions (872.4, 914.5, 1428.7, 1495.7) found overexpressed in melanoma tissues. Two peptides (*m*/*z* 976.4, 1998.7) derived from actin cytoplasmic 1 protein were also high expressed in melanoma samples. Likewise, histone H2B type 1 (*m*/*z* 816.4), histone H4 (*m*/*z* 1325.7), and stress-70 protein mitochondrial (*m*/*z* 958.5) were highly abundant in melanoma tissues. MS/MS analysis of the discriminant ions at *m*/*z* 631.4, 901.3, 957.6, 1489.6, 1490.8, and 3052.6 did not yield significant sequence matches (MASCOT score < 30) due to their low intensities.

## 3. Discussion

Most of the melanocytic lesions can be classified by conventional histological investigation alone. Some melanocytic lesions require application of immunohistological techniques [6,7,8] or even molecular biological methods [29]. Some lesions cannot be classified definitively [10,30]. Among the immunohistological markers, S100 protein, HMB-45, MIB, PRAME, and p16 [6,8] are widely used. Other discriminating immunohistochemical markers include BRCA1-associated protein-1 (BAP-1), SOX10, and tyrosinase [31,32]. Loss of BAP-1 expression in malignant melanoma may be also helpful in discriminating benign from malignant melanocytic lesions [31]. HMB-45, SOX10, and tyrosinase, but not melan-A, proved to differentiate the nevi from malignant melanomas successfully, with high specificity [32]. In situ hybridization assays for the differential diagnosis of nevus versus melanoma is now widely used in dermatopathology laboratories. A four-probe FISH assay targeting 6p25 (RREB1), 6q23 (MYB), Cep6 (centromere 6), and 11q13 (CCND1) could discriminate between histologically unequivocal melanomas and benign nevi with a sensitivity of 86.7% and specificity of 95.4% [11]. These sets were later modified as 9p21, 6p25, 11q13, and 8q24, which showed improved discriminatory power in differentiating melanomas from nevi [9]. The value of CCND1 amplification by FISH as a diagnostic marker for histologically undetermined early acral melanoma in situ was emphasized by Cho-Vega, J.H, et al. [10]. Even today, with the next-generation sequencing techniques, there is an overlap of mutations in melanocytic nevi and malignant melanomas [33].

Prior studies demonstrated that imaging mass spectrometry technology was successfully applied to differentiate melanocytic lesions (spitz nevus from malignant melanoma showing spitzoid features) [34] or for prognostic information [27]. Since then, there has been advancement in the instrumentation and refinement of the sample processing protocols, especially towards to reproducibility and standardization [27,35,36]. Here, we used a proteomics-based IMS approach, with a new generation platform for the analysis of FFPE tissues. Prior to the classification study, we carried out experiments to implement standardization, and increase the quality, accuracy, and reproducibility of the mass spectral data for these specific tissue entities. We observed that the antigen retrieval temperature at 110 °C produced increased peak intensities when compared to the condition at 95 °C (Figures S1 and S2). When we tested two different trypsin deposition methods (wet and dry), we did not observe considerable differences in regard to intensity; thus, we opted for the wet condition, as it was the fastest deposition method. In addition, off-tissue areas, including matrix and autolysis tryptic molecules, were analyzed alongside the tissue samples. Off-tissues were examined to ensure that no contamination originating from background signals (e.g., matrix clusters and trypsin) could be considered in the data analysis. Finally, the efficacy of the tryptic digestion was also evaluated by the presence of one very common trypsin autolysis product (*m*/*z* 842.5). These quality assurance parameters were carefully controlled and considered in the analysis of all tissues, ensuring a robust and reproducible methodology.

PCA is usually used as a processing step prior to the application of more advanced data analysis algorithms. Thus, an initial unsupervised clustering analysis was performed using PCA to obtain information about the variability and heterogeneity of the spectra dataset. As a result, the largest variance was described in the first three principal components accounting of 70%, which could spread the data in two groups, corresponding to melanoma and nevi. To achieve dimensionality reduction, UMAP analysis was performed, which separated data in two main clusters corresponding to melanoma and melanocytic nevi.

We have demonstrated a high accuracy (98%) for a supervised LDA classifier distinguishing malignant melanoma and melanocytic nevi on independent data. When the model was tested on a separated dataset, its performance was slightly lower in the range of 91%–95% in terms of sensitivity and specificity, and 93% of overall accuracy. This is likely due to the small number of samples used (90 patients) compared to the number of samples used in the training set (113 patients). Furthermore, good performance of the LDA classifier was also achieved with a smaller number of features (18 instead of 187 peptides), reaching a comparable accuracy of 93% and 92% in the training and testing set, respectively. In view of this, a decreased number of peptides did not improve the classification accuracy; as a matter of fact, all 187 peptides were selected based on the area under the ROC curve (AUC) ≥ 0.7, thus having a good discrimination quality. In this regard, a proteomics-based IMS classification approach could help in the evaluation of melanocytic lesions as a rapid, sensitive, specific, and especially unbiased method.

Most of the differentially expressed peptides were observed at higher intensity in the melanoma specimens. We detected vimentin as an upregulated protein in the melanoma samples. Numerous studies relating to proteomics have shown that vimentin is associated with tumor growth and metastasis in multiple malignancies [37,38,39]. Vimentin is a major cytoskeletal component of mesenchymal cells, and it is often used as a marker for the epithelial–mesenchymal transition during both normal development and metastatic progression. Thus, overexpression of vimentin in tumors not only serves as a diagnostic marker, but may also act as a predictor of metastatic potential [40].

The overexpression of actin in melanoma compared to nevi is ascribed to the active remodeling of a cell’s actin cytoskeleton, essential for tumor cell invasion [41]. These results correspond well with a previous analysis on cutaneous melanoma cells, where altered expression of both actin and vimentin revealed increased the cellular elasticity with as a consequence an increase in the migratory properties of melanoma cells [42]. Differences in the expression of vimentin and actin in spitz nevi and spitzoid malignant melanoma were detected by mass spectrometry in a previous study, but the results could not be validated by immunohistochemistry [43]. This could be due to the limited sensitivity of antibodies against actin and vimentin. In this regard, it is important to note that mass spectrometry can also detect actin isoforms. In mammals, actin is represented by six isoforms that are 95–99% identical to each other; however, the six actin genes have vastly different functions in vivo, and importance of specific amino acid sequences for each actin isoform are not well understood [44]. Although actin is still associated mainly with the cytoskeleton, it has been shown that actin is also important inside the cell nucleus. Actin has been linked to many gene expression processes, from gene activation to chromatin remodeling, but also to maintenance of genomic integrity and intranuclear movement of chromosomes and chromosomal loci [45]. Common high-confidence interactions highlight the role of actin in chromatin-remodeling complexes and identify the histone-modifying complex human Ada-Two-A-containing (hATAC) as a novel actin-containing nuclear complex [46].

Increased intensity of histones H2B and H4 in the malignant melanoma is in line with other studies where dysregulation of the histone modification system contributed to the loss of tumor suppressors or enhanced proliferative capacity in melanoma as well as in other cancers [47,48]. Transcriptional and histone modification signatures that may be molecular events driving melanoma progression and metastasis have been revealed, which can aid in the identification of novel prognostic genes and drug targets for treating the disease [49]. It has been shown that H4-methylation was different in the more aggressive compared to the less aggressive melanoma cell line. Application of immunohistochemistry of histone modifications may increase the accuracy and confidence in the diagnosis of melanoma [50].

We found decreased collagen levels in malignant melanoma compared to nevi. This could represent a structural rearrangement during the progression from benign to malignant lesions. It has been shown that blood-based biomarkers reflecting excessive type III collagen were associated with worse survival after PD-1 inhibition in metastatic melanoma [51].

Interestingly, 3-methyladenine DNA glycosylases, a DNA base repair enzyme, was low expressed in melanoma compared to nevi tissues. This suggests a deficiency in removing damaged bases from the genome that can lead to mutations, or breaks in DNA during replication, and thus, contribute to the development of cancer [52].

A limitation of this study is the sample size. Further confirmation of the classification analysis requires a larger group of cases possibly from different counties, for the transition from discovery-based approaches to standard clinical practices. Nevertheless, our findings strongly demonstrate the effectiveness of IMS to discriminate melanoma from melanocytic nevi.

## 4. Materials and Methods

### 4.1. Tissue Collection and Preparation

The tissue cohort analyzed in this study included melanocytic nevi (*n* = 102) and malignant melanomas (*n* = 101). The histopathological features of each sample were reviewed by an experienced pathologist (J.K.) to confirm diagnosis and tumor content. This study was approved by the Ethical Committees of the University of Heidelberg (#315-20).

### 4.2. On-Tissue Digestion and Matrix Application

Tissues were processed according to standard and automated protocols. Briefly, tissue samples were fixed for 12–24 h in 10% neutral buffered formalin, dehydrated in graded ethanol, cleared in xylene, and embedded in paraffin. FFPE tissues were sectioned at room temperature using a microtome at a thickness of 3 µm. The sections were then mounted onto conductive indium tin oxide (ITO)-coated glass slides (Bruker Daltonik, Bremen, Germany) pre-coated with poly-L-lysine (Sigma Aldrich Chemie, Taufkirchen, Germany) solution (0.1% *v/v* in water), dried overnight at 37 °C, and then stored at room temperature until analysis. The total samples set spanned 61 ITO slides containing over 200 tissue sections. Serial sections were also collected for each tissue and stained with HE for histological examination.

FFPE sections were subjected to paraffin removal with xylene (100%, twice for 5 min), isopropanol wash (100% for 5 min), graded ethanol washes (100%, 95%, 70%, and 50% for 5 min each), and purified water (5 s). All solvents are purchased from Fisher Scientific, Schwerte, Germany). Dewax tissue sections were then placed directly into 10 mM Tris (Sigma Aldrich Chemie, Taufkirchen, Germany) buffer pH 9.0 for antigen retrieval procedure, which were accomplished by pressure heating the slides for 20 min at 110 °C using a Biocare Medical decloaking chamber (ZITOMED Systems GmbH, Berlin, Germany). For a comparative sample preparation protocol that we investigated in a preliminary study, antigen retrieval was performed at 95 °C for 20 min. The slides were cooled to room temperature using a series of five washings in water [53]. Proteins were on-tissue digested into peptide fragments using the procedure outlined by Ly et al. [27]. Briefly, trypsin (Promega, Mannheim, Germany) was diluted in 20 mM ammonium bicarbonate (Sigma Aldrich Chemie) to reach a concentration of 0.025 µg/µL. The trypsin solution was immediately deposited across the tissues by an automatic reagent sprayer (TM-Sprayer, HTX Technologies, Chapel Hill, NC, USA) in eight layers using the following parameters: temperature of 30 °C, 0.03 mL/min flow rate, and 750 mm/min velocity. The trypsin spraying method was another parameter that we wanted to evaluate in order to improve data quality. Thus, in our test and comparative experiments, trypsin was sprayed in a dry mode, including 16 passes with the following parameters: temperature of 30 °C, 0.015 mL/min flow rate, and 750 mm/min velocity. Slides were then placed inside the digestion chamber, prepared with saturated potassium sulphate solution (Carl Roth Karlsruhe, Germany) and incubated at 50 °C for 2 h. After digestion, tissues were sprayed with four layers of matrix solution made from 10 mg/mL α-cyano-4-hydroxycinnamic acid (Bruker Daltonik, Billerica, MA, USA) in 70% acetonitrile, 1% trifluoroacetic acid (Fisher Scientific) using the same sprayer devise with the following parameters: temperature of 75 °C, 0.120 mL/min flow rate, and 1200 mm/min velocity.

### 4.3. MALDI Imaging Mass Spectrometry (IMS) Profiling

Direct tissue mass spectral analysis was carried out through a “profiling” approach where experiments were designed to make comparisons between representative areas on small areas of tissue. Thus, only specific locations within the tissue sections were analyzed that correlated with the histological annotation of their corresponding consecutive sections. The MALDI mass spectra presented here are generated in an automated mode using a rapifleX MALDI Tissue-typer mass spectrometer (Bruker Daltonik) by averaging signals from 500 laser pulses per matrix position. For every measurement, the instrument was externally calibrated using the Peptide Calibration Standard II (Bruker Daltonik). Following MALDI analysis, the matrix was removed from the samples with 100% ethanol, the slides were then stained with HE and scanned with 40× objective magnification using two slide scanners (3DHISTECH Ltd., Budapest, Hungary, and Aperio AT2 slide scanner, Leica Biosystems, Wetzlar, Germany).

### 4.4. Data Analysis

Mass spectra were subjected to a series of processing steps to prepare data for statistical analysis. The analytical goals of profiling experiments were two-fold: (1) the classification of samples into two classes (melanoma/nevi) and (2) the identification of potential biomarkers characteristic to each class. The preprocessing steps included baseline subtraction using the Top Hat algorithm performed by flexImaging software (Bruker Daltonik), and spectra normalization, which was performed by scaling spectra according to the total measured ion current (TIC) using SCiLS lab software (Bruker Daltonik). All spectra were further imported into R statistical software [28] and processed for mass shift analysis and alignment procedure [54,55].

We performed an initial unsupervised clustering using Uniform Manifold Approximation and Projection (UMAP) and principal component analysis (PCA) to explore data and maximize the variance. UMAP analysis was done in R statistical software (v. 4.1.0) with the help of UMAP package (v.0.2.7.0). Perplexity was set to 30; other standard parameters were not changed. The UMAP plot was created with the ggpubr package (v. 0.4.0). PCA was conducted on all individual spectra from the annotated regions with weak denoising, and unit variance scaling was dnoe by using SCiLS lab. Annotated HE images were overlaid with MALDI data to correlate molecular MS data with histological entities in the same tissue section. Thus, spectra from each annotated region were grouped together for each patient and for each melanocytic type. The cohort was then randomly separated into a training set, including 56 malignant melanoma and 57 melanocytic nevi, used to build a classification model, and a testing set, including 45 malignant melanoma and 45 melanocytic nevi, to validate the model. The selection of the monoisotopic relevant peaks (*n* = 187) in the spectra averages was assessed through the Receiver Operating Characteristic (ROC) analysis to create a peak list which was further processed for spectra classification. The classification study was accomplished using the linear discriminant analysis (LDA) algorithm supported by SCiLS lab software.

### 4.5. Protein Identification

*M/z* ion peptides that were significantly expressed by the specific tissue phenotype were identified using a rapifleX MALDI MS/MS Tissue-typer mass spectrometer (Bruker Daltonik) directly from the digested tissues. Sample preparation for MALDI MS/MS was identical to that described above. The mass spectrometer was first operated in reflectron mode in the range of *m*/*z* 600–3000 to collect a full scan mass spectrum from the tissue regions of interest to confirm the presence of the peaks of interest and check the parent ion masses. For MS/MS analysis, the instrument was operated in Lift mode with the following operating parameters: ion source voltage-1 = 20 kV, ion source voltage-2 = 19.45 kV, reflector voltage-1 = 23.8 kV, reflector voltage-2 = 1.79 kV, reflector voltage-3 = 9.85 kV, reflector detector voltage = 2.64 kV, laser repetition rate = 5000 Hz, sample rate = 1.6 ns, realtime smooth = medium. The peptide precursor of interest was selected and fragmented using collision-induced dissociation (CID) by increasing the laser fluence to generate high fragment ion yields. Each MS/MS spectrum was processed with FlexAnalysis 4.0 (Bruker Daltonik) for baseline subtraction using TopHat, peak peaking using the SNAP algorithm and with a signal/noise threshold = 3. The resulting spectrum fragmentation patterns were searched against the MASCOT database (Mascot 2.7.0.5 SwissProt_2020_06.fasta) for corresponding sequence patterns. Parent and fragment ion tolerances were set at 200 ppm and ± 0.3 Da, respectively. Up to one missed cleavage was included, and the variable modifications allowed were: Arg-loss (C-term R), protein N terminus acetylation, histidine, methionine, and proline oxidation. UniProtKB/Swiss-Prot (www.uniprot.org, accessed on 17 May 2021) was used as the reference database (human taxonomy). Trypsin was selected as the proteolytic enzyme.

## 5. Conclusions

Although recent studies have contributed greatly to the development of melanoma markers, up to now, there were no molecular biomarkers able to distinguish malignant from benign nevi. We demonstrate that IMS, as an objective method using molecular biomarkers, is a promising diagnostic tool to classify malignant melanoma from benign melanocytic nevi. We anticipate that the application of IMS will offer great potential for the improved characterization of clinical tissues, and suggest biomarker candidates that can be routinely used in clinical practice.

## Figures and Tables

**Table 1:** Classification results of melanoma and nevi samples through MALDI MSI-based proteomic analysis.

Peptide Signature	Cohort	Tissue Type	*n* Patients	Correct Classification ^1^	Incorrect Classification	Overall Accuracy
187 Peptides	Training	Melanoma	56	54	2	98%
		Nevus	57	57	0	
	Testing	Melanoma	45	41	4	93%
		Nevus	45	43	2	
18 Peptides	Training	Melanoma	56	49	7	93%
		Nevus	57	56	1	
	Testing	Melanoma	45	39	6	92%
		Nevus	45	44	1	

^1^ In agreement with the diagnosis made by pathologists.

## Data Availability

The data are available from Rita Casadonte upon reasonable request.

## Supplementary Materials

The following are available online at https://www.mdpi.com/article/10.3390/cancers13133197/s1, Figure S1: Evaluation of the peptide peaks in regards to their intensity, Figure S2: Result of different antigen retrieval temperature and trypsin deposition protocols obtained from two melanocytic areas of two individual tissues, Table S1: List of peptides identified by on-tissue MALDI MS/MS.

## References

[1] Siegel R.L., Miller K.D., Fuchs H.E., Jemal A. (2021). Cancer Statistics, 2021. CA Cancer J. Clin..

[2] Bevona C., Goggins W., Quinn T., Fullerton J., Tsao H. (2003). Cutaneous melanomas associated with nevi. Arch. Dermatol..

[3] Tsao H., Bevona C., Goggins W., Quinn T. (2003). The transformation rate of moles (melanocytic nevi) into cutaneous melanoma: A population-based estimate. Arch. Dermatol..

[4] Gruber S.B., Barnhill R.L., Stenn K.S., Roush G.C. (1989). Nevomelanocytic proliferations in association with cutaneous malignant melanoma: A multivariate analysis. J. Am. Acad. Dermatol..

[5] Farmer E.R., Gonin R., Hanna M.P. (1996). Discordance in the histopathologic diagnosis of melanoma and melanocytic nevi between expert pathologists. Hum. Pathol..

[6] Garola R., Singh V. (2019). Utility of p16-Ki-67-HMB45 score in sorting benign from malignant Spitz tumors. Pathol. Res. Pract..

[7] Ritter A., Tronnier M., Vaske B., Mitteldorf C. (2018). Reevaluation of established and new criteria in differential diagnosis of Spitz nevus and melanoma. Arch. Dermatol. Res..

[8] See S.H.C., Finkelman B.S., Yeldandi A.V. (2020). The diagnostic utility of PRAME and p16 in distinguishing nodal nevi from nodal metastatic melanoma. Pathol. Res. Pract..

[9] Gerami P., Li G., Pouryazdanparast P., Blondin B., Beilfuss B., Slenk C., Du J., Guitart J., Jewell S., Pestova K. (2012). A highly specific and discriminatory FISH assay for distinguishing between benign and malignant melanocytic neoplasms. Am. J. Surg. Pathol..

[10] Cho-Vega J.H., Cao T., Ledon J., Moller M., Avisar E., Elgart G., Tan J.H., Fan Y.S., Grichnik J.M. (2021). Diagnostic application of cyclin D1 fluorescent in situ hybridization for histologically undetermined early lesions of acral melanoma in situ: A case series. Ann. Diagn. Pathol..

[11] Pouryazdanparast P., Newman M., Mafee M., Haghighat Z., Guitart J., Gerami P. (2009). Distinguishing epithelioid blue nevus from blue nevus-like cutaneous melanoma metastasis using fluorescence in situ hybridization. Am. J. Surg. Pathol..

[12] Yanovich G., Agmon H., Harel M., Sonnenblick A., Peretz T., Geiger T. (2018). Clinical Proteomics of Breast Cancer Reveals a Novel Layer of Breast Cancer Classification. Cancer Res..

[13] Guo T., Li L., Zhong Q., Rupp N.J., Charmpi K., Wong C.E., Wagner U., Rueschoff J.H., Jochum W., Fankhauser C.D. (2018). Multi-region proteome analysis quantifies spatial heterogeneity of prostate tissue biomarkers. Life Sci. Alliance.

[14] Timms J.F., Hale O.J., Cramer R. (2016). Advances in mass spectrometry-based cancer research and analysis: From cancer proteomics to clinical diagnostics. Expert Rev. Proteom..

[15] Longuespee R., Casadonte R., Kriegsmann M., Pottier C., Picard de Muller G., Delvenne P., Kriegsmann J., De Pauw E. (2016). MALDI mass spectrometry imaging: A cutting-edge tool for fundamental and clinical histopathology. Proteom. Clin. Appl..

[16] Rauser S., Marquardt C., Balluff B., Deininger S.O., Albers C., Belau E., Hartmer R., Suckau D., Specht K., Ebert M.P. (2010). Classification of HER2 receptor status in breast cancer tissues by MALDI imaging mass spectrometry. J. Proteome Res..

[17] Schwamborn K., Krieg R.C., Reska M., Jakse G., Knuechel R., Wellmann A. (2007). Identifying prostate carcinoma by MALDI-Imaging. Int. J. Mol. Med..

[18] Kriegsmann M., Casadonte R., Kriegsmann J., Dienemann H., Schirmacher P., Hendrik Kobarg J., Schwamborn K., Stenzinger A., Warth A., Weichert W. (2016). Reliable Entity Subtyping in Non-small Cell Lung Cancer by Matrix-assisted Laser Desorption/Ionization Imaging Mass Spectrometry on Formalin-fixed Paraffin-embedded Tissue Specimens. Mol. Cell. Proteom..

[19] Kriegsmann M., Casadonte R., Maurer N., Stoehr C., Erlmeier F., Moch H., Junker K., Zgorzelski C., Weichert W., Schwamborn K. (2020). Mass Spectrometry Imaging Differentiates Chromophobe Renal Cell Carcinoma and Renal Oncocytoma with High Accuracy. J. Cancer.

[20] Casadonte R., Kriegsmann M., Perren A., Baretton G., Deininger S.O., Kriegsmann K., Welsch T., Pilarsky C., Kriegsmann J. (2019). Development of a Class Prediction Model to Discriminate Pancreatic Ductal Adenocarcinoma from Pancreatic Neuroendocrine Tumor by MALDI Mass Spectrometry Imaging. Proteom. Clin. Appl..

[21] Van de Plas R., Yang J., Spraggins J., Caprioli R.M. (2015). Image fusion of mass spectrometry and microscopy: A multimodality paradigm for molecular tissue mapping. Nat. Methods.

[22] Welinder C., Pawłowski K., Sugihara Y., Yakovleva M., Jönsson G., Ingvar C., Lundgren L., Baldetorp B., Olsson H., Rezeli M. (2015). A protein deep sequencing evaluation of metastatic melanoma tissues. PLoS ONE.

[23] Azimi A., Kaufman K.L., Kim J., Ali M., Mann G.J., Fernandez-Penas P. (2020). Proteomics: An emerging approach for the diagnosis and classification of cutaneous squamous cell carcinoma and its precursors. J. Dermatol. Sci..

[24] Matharoo-Ball B., Ratcliffe L., Lancashire L., Ugurel S., Miles A.K., Weston D.J., Rees R., Schadendorf D., Ball G., Creaser C.S. (2007). Diagnostic biomarkers differentiating metastatic melanoma patients from healthy controls identified by an integrated MALDI-TOF mass spectrometry/bioinformatic approach. Proteom. Clin. Appl..

[25] Mian S., Ugurel S., Parkinson E., Schlenzka I., Dryden I., Lancashire L., Ball G., Creaser C., Rees R., Schadendorf D. (2005). Serum proteomic fingerprinting discriminates between clinical stages and predicts disease progression in melanoma patients. J. Clin. Oncol..

[26] Hardesty W.M., Kelley M.C., Mi D., Low R.L., Caprioli R.M. (2011). Protein signatures for survival and recurrence in metastatic melanoma. J. Proteom..

[27] Ly A., Longuespée R., Casadonte R., Wandernoth P., Schwamborn K., Bollwein C., Marsching C., Kriegsmann K., Hopf C., Weichert W. (2019). Site-to-Site Reproducibility and Spatial Resolution in MALDI-MSI of Peptides from Formalin-Fixed Paraffin-Embedded Samples. Proteom. Clin. Appl..

[28] R Core Team (2019). R: A Language and Environment for Statistical Computing.

[29] Kerl K., Palmedo G., Wiesner T., Mentzel T., Rutten A., Scharer L., Paredes B., Hantschke M., Kutzner H. (2012). A proposal for improving multicolor FISH sensitivity in the diagnosis of malignant melanoma using new combined criteria. Am. J. Dermatopathol..

[30] Ebbelaar C.F., Jansen A.M.L., Bloem L.T., Blokx W.A.M. (2021). Genome-wide copy number variations as molecular diagnostic tool for cutaneous intermediate melanocytic lesions: A systematic review and individual patient data meta-analysis. Virchows Arch..

[31] Chen P.L., Neishaboori N., Tetzlaff M.T., Chen W.S., Aung P.P., Curry J.L., Nagarajan P., Ivan D., Hwu W.J., Prieto V.G. (2020). BAP-1 Expression Status by Immunohistochemistry in Cellular Blue Nevus and Blue Nevus-like Melanoma. Am. J. Dermatopathol..

[32] Beleaua M.A., Jung I., Braicu C., Milutin D., Gurzu S. (2021). SOX11, SOX10 and MITF Gene Interaction: A Possible Diagnostic Tool in Malignant Melanoma. Life.

[33] Lozada J.R., Geyer F.C., Selenica P., Brown D., Alemar B., Merghoub T., Berger M.F., Busam K.J., Halpern A.C., Weigelt B. (2019). Massively parallel sequencing analysis of benign melanocytic naevi. Histopathology.

[34] Lazova R., Seeley E.H., Keenan M., Gueorguieva R., Caprioli R.M. (2012). Imaging mass spectrometry--a new and promising method to differentiate Spitz nevi from Spitzoid malignant melanomas. Am. J. Dermatopathol..

[35] Hermann J., Noels H., Theelen W., Lellig M., Orth-Alampour S., Boor P., Jankowski V., Jankowski J. (2020). Sample preparation of formalin-fixed paraffin-embedded tissue sections for MALDI-mass spectrometry imaging. Anal. Bioanal. Chem..

[36] Vaysse P.M., Heeren R.M.A., Porta T., Balluff B. (2017). Mass spectrometry imaging for clinical research—Latest developments, applications, and current limitations. Analyst.

[37] Korsching E., Packeisen J., Liedtke C., Hungermann D., Wülfing P., van Diest P.J., Brandt B., Boecker W., Buerger H. (2005). The origin of vimentin expression in invasive breast cancer: Epithelial-mesenchymal transition, myoepithelial histogenesis or histogenesis from progenitor cells with bilinear differentiation potential?. J. Pathol..

[38] Upton M.P., Hirohashi S., Tome Y., Miyazawa N., Suemasu K., Shimosato Y. (1986). Expression of vimentin in surgically resected adenocarcinomas and large cell carcinomas of lung. Am. J. Surg. Pathol..

[39] Satelli A., Li S. (2011). Vimentin in cancer and its potential as a molecular target for cancer therapy. Cell. Mol. Life Sci..

[40] Li M., Zhang B., Sun B., Wang X., Ban X., Sun T., Liu Z., Zhao X. (2010). A novel function for vimentin: The potential biomarker for predicting melanoma hematogenous metastasis. J. Exp. Clin. Cancer Res..

[41] Pawlak G., Helfman D.M. (2001). Cytoskeletal changes in cell transformation and tumorigenesis. Curr. Opin. Genet. Dev..

[42] Jasińska-Konior K., Wiecheć O., Sarna M., Panek A., Swakoń J., Michalik M., Urbańska K., Elas M. (2019). Increased elasticity of melanoma cells after low-LET proton beam due to actin cytoskeleton rearrangements. Sci. Rep..

[43] Alomari A.K., Klump V., Neumeister V., Ariyan S., Narayan D., Lazova R. (2015). Comparison of the expression of vimentin and actin in spitz nevi and spitzoid malignant melanomas. Am. J. Dermatopathol..

[44] Kashina A.S. (2020). Regulation of actin isoforms in cellular and developmental processes. Semin. Cell Dev. Biol..

[45] Viita T., Vartiainen M.K. (2017). From Cytoskeleton to Gene Expression: Actin in the Nucleus. Handb. Exp. Pharmacol..

[46] Viita T., Kyheroinen S., Prajapati B., Virtanen J., Frilander M.J., Varjosalo M., Vartiainen M.K. (2019). Nuclear actin interactome analysis links actin to KAT14 histone acetyl transferase and mRNA splicing. J. Cell Sci..

[47] Kulis M., Esteller M. (2010). DNA methylation and cancer. Adv. Genet..

[48] Molden R.C., Bhanu N.V., LeRoy G., Arnaudo A.M., Garcia B.A. (2015). Multi-faceted quantitative proteomics analysis of histone H2B isoforms and their modifications. Epigenetics Chromatin.

[49] Azevedo H., Pessoa G.C., de Luna Vitorino F.N., Nsengimana J., Newton-Bishop J., Reis E.M., da Cunha J.P.C., Jasiulionis M.G. (2020). Gene co-expression and histone modification signatures are associated with melanoma progression, epithelial-to-mesenchymal transition, and metastasis. Clin. Epigenetics.

[50] Davis L.E., Shalin S.C., Tackett A.J. (2020). Utility of histone H3K27me3 and H4K20me as diagnostic indicators of melanoma. Melanoma Res..

[51] Hurkmans D.P., Jensen C., Koolen S.L.W., Aerts J., Karsdal M.A., Mathijssen R.H.J., Willumsen N. (2020). Blood-based extracellular matrix biomarkers are correlated with clinical outcome after PD-1 inhibition in patients with metastatic melanoma. J. Immunother. Cancer.

[52] Osorio A., Milne R.L., Kuchenbaecker K., Vaclová T., Pita G., Alonso R., Peterlongo P., Blanco I., de la Hoya M., Duran M. (2014). DNA glycosylases involved in base excision repair may be associated with cancer risk in BRCA1 and BRCA2 mutation carriers. PLoS Genet..

[53] Casadonte R., Caprioli R.M. (2011). Proteomic analysis of formalin-fixed paraffin-embedded tissue by MALDI imaging mass spectrometry. Nat. Protoc..

[54] Boskamp T., Lachmund D., Casadonte R., Hauberg-Lotte L., Kobarg J.H., Kriegsmann J., Maass P. (2020). Using the Chemical Noise Background in MALDI Mass Spectrometry Imaging for Mass Alignment and Calibration. Anal. Chem..

[55] Boskamp T., Casadonte R., Hauberg-Lotte L., Deininger S., Kriegsmann J., Maass P. (2021). Cross-normalization of MALDI mass spectrometry imaging data improves site-to-site reproducibility. Anal. Chem..

