# Supplementary material for: Imaging Mass Spectrometry-Based Proteomic Analysis to Differentiate Melanocytic Nevi and Malignant Melanoma
Source: Cancers (Basel). 2021 Jun 26;13(13):3197. doi: 10.3390/cancers13133197 (PMC8267712; doi:10.3390/cancers13133197)
Supplement: Supplementary file 1 [file cancers-13-03197-s001.zip › Supplementary Materials_v5 final.pdf]

Supplementary Materials: Imaging Mass Spectrometry-Based Proteomic Analysis to Differentiate Melanocytic Nevi and Malignant Melanoma

Rita Casadonte, Mark Kriegsmann, Katharina Kriegsmann, Isabella Hauk, Rolf Rüdiger Meliß, Cornelia S.L. Müller, and Jörg Kriegsmann

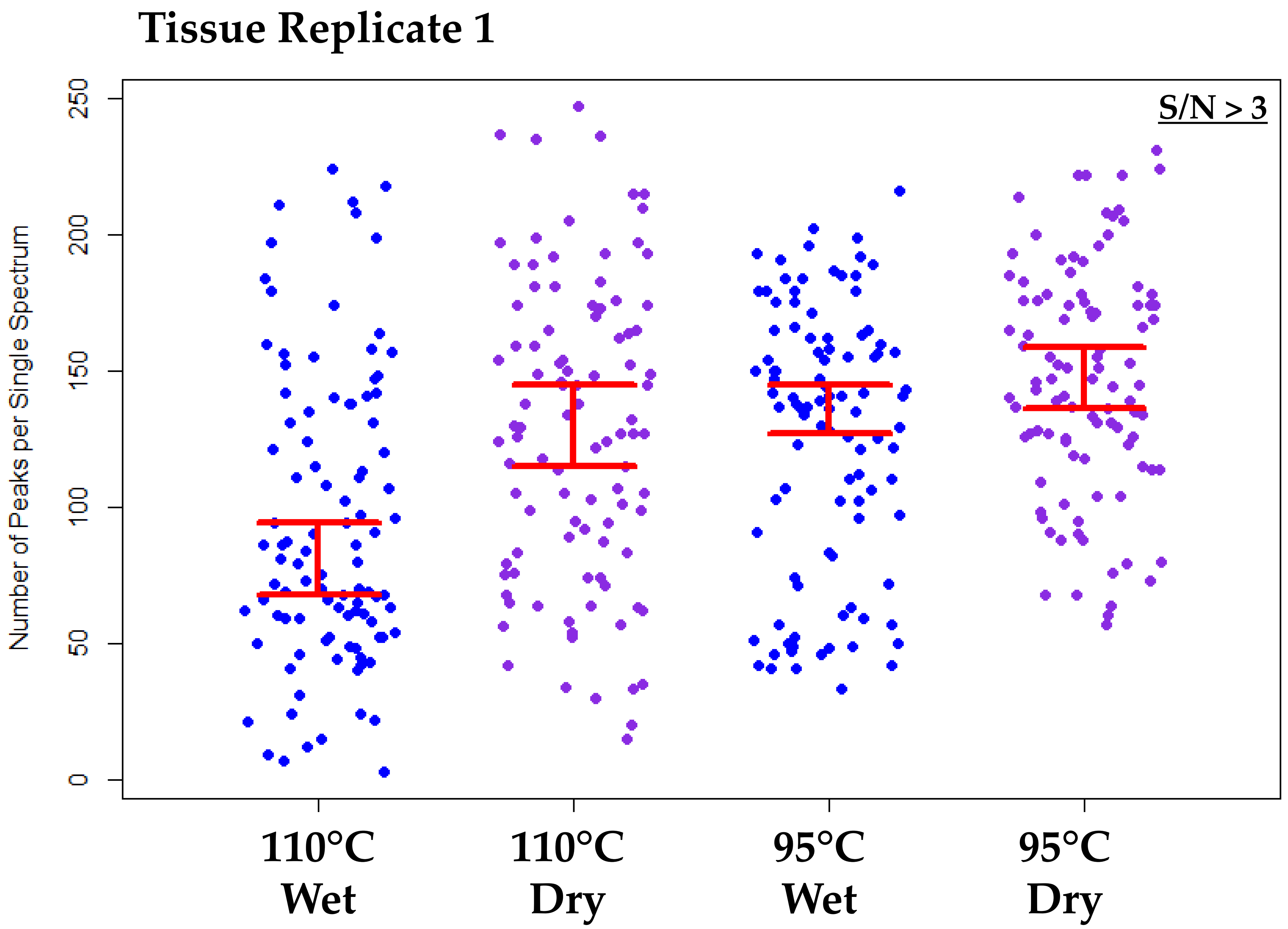

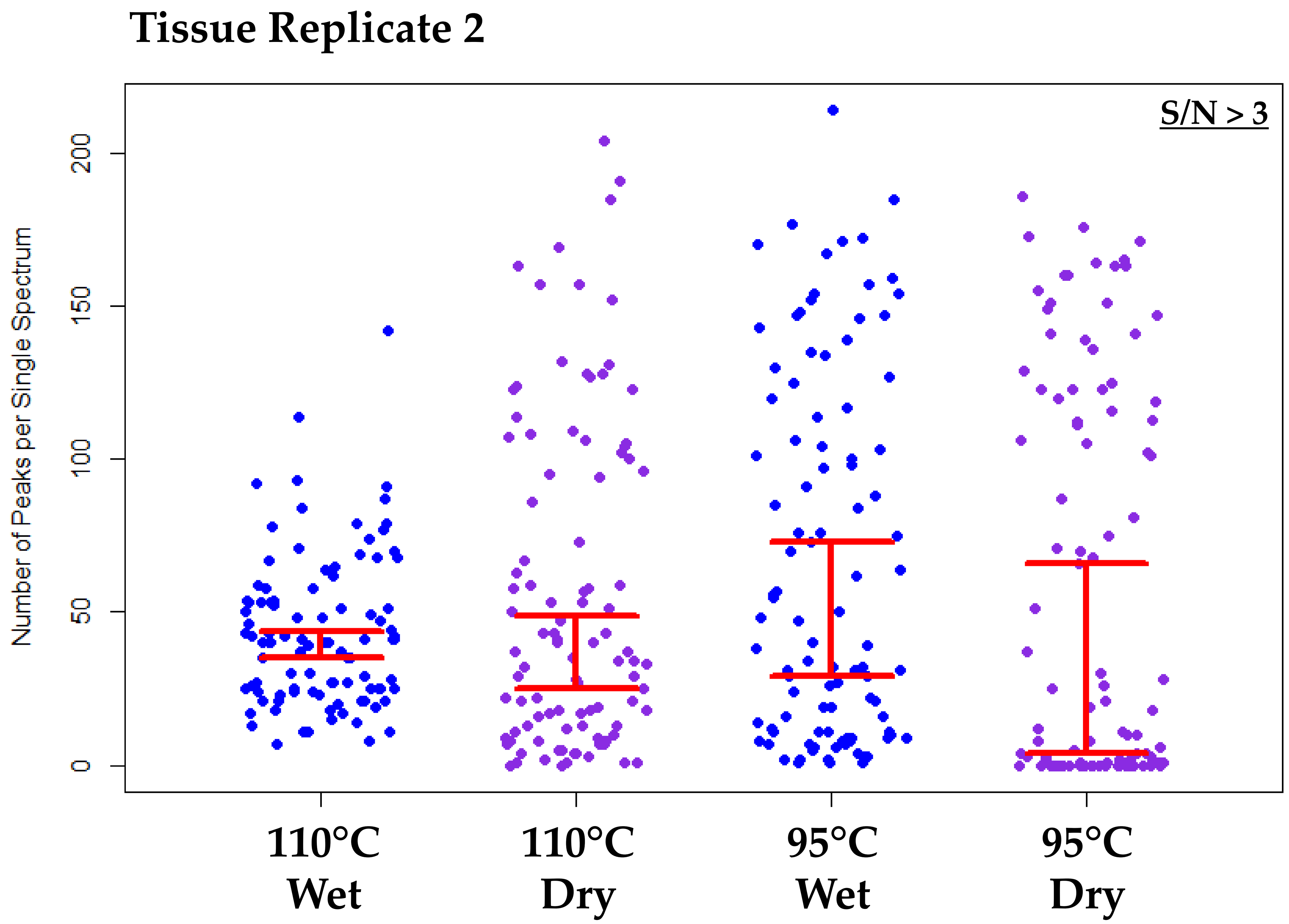

Method	110°C-Wet	110°C-Dry	95°C-Wet	95°C-Dry
**median total peak count (S/N>1)**	109.5	194.5	160.5	171.5
**peak count with S/N > 3**	80.5	128	137.5	145.5
**peak count with m/z > 1500**	21	43.5	5	14.5
**peak count with m/z > 2000**	9	14.5	0	1

Method	110°C-Wet	110°C-Dry	95°C-Wet	95°C-Dry
**median total peak count (S/N>1)**	51	44	48.5	17
**peak count with S/N > 3**	40	36	43.5	15
**peak count with m/z > 1500**	6	5	8.0	0
**peak count with m/z > 2000**	1	1	2.0	0

Figure S1. Evaluation of the peptide peaks in regards to their intensity: Graphical plots showing comparison of the median total peak count, per individual spectra with a signal-to-noise (S/N)>3, of all preliminary experiments testing antigen retrieval temperature (95 °C, 110 °C) and trypsin deposition (wet, dry) approaches in a melanoma tissue. All experiments were performed in duplicate (graphical images up left, and right). Peak count comparison across different experiments with different S/N (>1, >3) and selected mass-to-charge ratio (*m/z*) range (>1500, >2000) is shown in the table below for both tissue replicates.

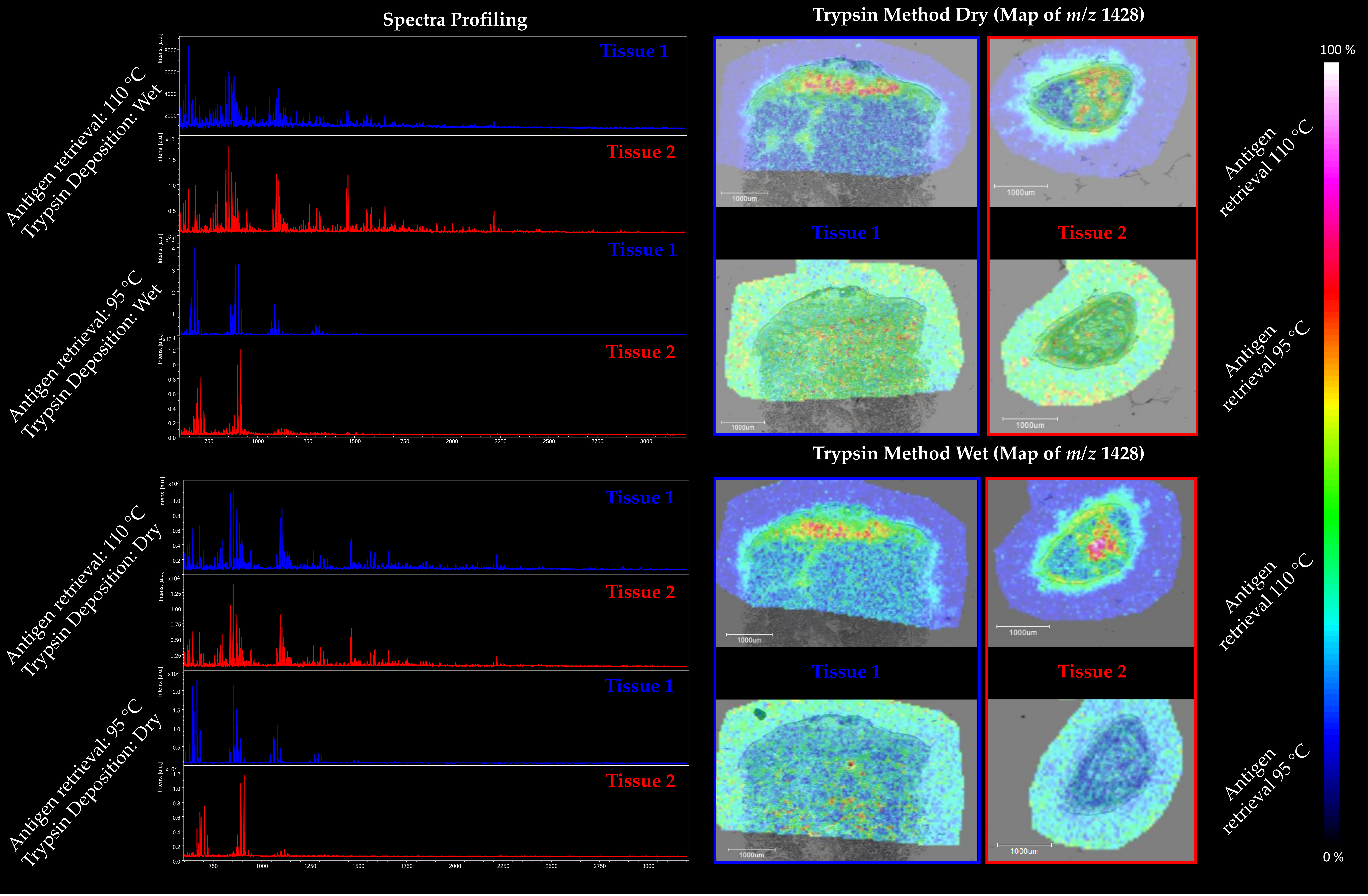

Figure S2. Result of different antigen retrieval temperature and trypsin deposition protocols obtained from two melanocytic areas of two individual tissues. Spectra profile comparison at different antigen retrieval temperature (95 °C, 110 °C) and trypsin deposition (wet, dry) procedures are shown in the left panel. Tissues threated with antigen retrieval temperature = 110 °C and with both trypsin deposition methods (wet, dry) showed higher peak intensity in comparison of antigen retrieval temperature = 95 °C. Mass distribution of a representative ion peptide (*m*/*z* 1428) between sample preparation experiments is shown on the right panel. High peak intensity is obtained in the experiments, either with the dry or wet trypsin deposition method, with antigen retrieval temperature = 110 °C. No evidence of delocalization across the sections was observed

Table S1. List of peptides identified by on-tissue MALDI MS/MS

Observed	Mr (expt)	Mr (calc)	Mass Error (ppm)	Mascot Score	Peptide Sequence / Modification	Protein Name	UniProt Accession Number
816.457	815.433	815.45	21	66	R.EIQTAVR.L	Histone H2B type 1	Q96A08
872.4110	871.4037	871.4035	0.2	77	R.SVSSSSYRR.M + Arg-loss (C-term R)	Vimentin	P08670
914.4570	913.4497	913.4505	- 0.87	59	R.SYVTTSTR.T	Vimentin	P08670
958.56	957.5527	957.4879	67	68	K.VLENAEGAR.T	Stress-70 protein, mitochondrial	P38646
976.4700	975.4627	975.4410	22.3	101	K.AGFAGDDAPR.A	Actin, cytoplasmic 1	P60709
1138.6	1137.5927	1137.5679	21	51	R.VGVGHAGEWAR.K	DNA-3-methyladenine glycosylase	P29372
1198.7100	1197.7027	1197.5150	157	53	K.DSYVGDEAQSK.R	Actin, cytoplasmic 1	P60709
1325.7700	1324.7627	1324.7463	12	69	R.DNIQGITKPAIR.R	Histone H4	P62805
1428.71	1427.7027	1427.7045	-1.23	62	R.SLYASSPGGVYATR.S	Vimentin	P08670
1495.7980	1494.7907	1494.7790	7	109	R.TYSLGSALRPSTSR.S	Vimentin	P08670
3068.48	3067.4727	3067.4094	20	168	R.GLPGPPGAPGPQGFQGPPGEPGEPGASGPMGPR.G + 2 Oxidation (P); [+31.9898 at P6]	Collagen alpha-1(I) chain	P02452

MALDI MS/MS, matrix-assisted laser desorption-ionization tandem mass spectrometry; Mr (expt), experimental relative molecular mass; Mr (calc), calculated relative molecular mass
